# Brain region and cell type-specific DNA methylation profiles in association with ADHD

**DOI:** 10.1038/s41598-025-18724-1

**Published:** 2025-10-08

**Authors:** Mandy Meijer, Gustavo Sudre, Kwangmi Ahn, Maggie Po Yuan Fu, Philip Shaw

**Affiliations:** 1 Department of Medical Genetics, Faculty of Medicine, University of British Columbia, 950 West 28th Avenue, Vancouver, BC V5Z4H4 Canada; 2 British Columbia Children’s Hospital Research Institute, University of British Columbia, Vancouver, BC Canada; 3 Centre for Molecular Medicine and Therapeutics, University of British Columbia, Vancouver, BC Canada; 4 Department of Child and Adolescent Psychiatry, Institute of Psychiatry, Psychology and Neuroscience, King’s College London, London, UK; 5 Social and Behavioral Research Branch, National Human Genome Research Institute, NIH, Bethesda, MD USA

**Keywords:** ADHD, DNA methylation, Epigenetics, Glutamatergic neurons, GABAergic neurons, Cell type, Bioinformatics, Methylation analysis, DNA methylation, ADHD

## Abstract

Previous studies identified DNA methylation (DNAm) associations with ADHD in peripheral tissue and the brain. Given that DNAm is highly cell type-specific, it is crucial to understand which cell types are driving the DNAm differences observed in ADHD. Here, we report the first brain cell type-specific epigenome-wide association study (EWAS) for ADHD (25 individuals with ADHD, 33 individuals without ADHD) in postmortem anterior cingulate cortex (ACC) and caudate nucleus (CN) based on epigenomic deconvolution. We identified distinct cell type-specific DNAm patterns in both brain regions. On the single site level, we identified significant associations with ADHD in microglia. We identified that in the ACC most differentially methylated regions (DMRs) differences were driven by glutamatergic neurons, whereas differences in the CN were mainly driven by GABAergic neurons. Enrichment of DMRs implicated genes involved in brain development, both in bulk and on the cell type-specific level. Genome-wide DNAm differences in microglia and GABAergic neurons were enriched in genetic risk variants for ADHD. Lastly, the results of EWAS were dependent on cell type reference panels used in statistical analyses. Altogether, these results could provide new insights into the molecular mechanisms underlying ADHD and considerations for EWAS in brain tissue.

**Supplementary Information:**

The online version contains supplementary material available at 10.1038/s41598-025-18724-1.

## Introduction

Epigenetic mechanisms could partially reflect the interplay between the genome and environment^1^; understanding epigenetic mechanisms can throw light into our understanding of childhood mental health conditions, such as ADHD^2^. DNA methylation is the most studied epigenetic modification, often through epigenome-wide association studies (EWAS). Previous EWAS of ADHD found modest associations with ADHD in childhood^3–7^ and adulthood^8–10^, with the most significant associations localizing in genes involved in neurodevelopment and lipid metabolism^4,5,9^. These studies have been mostly performed in peripheral tissues such as whole blood, cord blood, and saliva. Given that DNA methylation is highly tissue- and cell-type specific^11^, DNA methylation profiles in peripheral tissues do not necessarily reflect DNA methylation profiles in the brain. Therefore, to interpret DNA methylation findings as potential mechanisms underlying ADHD, DNA methylation studies in brain tissue are particularly insightful.

Recently, the first EWAS for ADHD diagnosis in brain tissue, specifically the anterior cingulate cortex (ACC) and caudate nucleus (CN) has been performed^12^. These brain regions are especially relevant for studying potential molecular mechanisms underlying ADHD given that they have been identified to show structural and functional differences in individuals with ADHD compared to individuals without ADHD based on (functional) MRI^13–15^, and are potentially contributing to ADHD aetiology. This first brain EWAS identified multiple differentially methylated regions (DMRs) implicated in neurodevelopment, specifically glial development^12^. These findings are in line with genetic enrichment studies implicating an association between ADHD and both neuronal cells^16–18^ and glial cells, including oligodendrocyte precursor cells^18,19^ and astrocytes^17,18^. Furthermore, a combined analysis of previous EWAS for ADHD and ADHD symptoms pointed towards the enrichment of DNA methylation sites in astrocyte marker genes^20^.

This earlier study did not consider the possibility that different brain cell types might show distinct DNA methylation profiles tied to ADHD diagnosis. It is possible to estimate cell type proportions present in bulk tissues^21,22^ through epigenomic deconvolution based on reference panels. These reference panels contain selected DNA methylation sites which have the most discriminative power of a predefined set of cell types based on external samples of a given tissue. Estimation of cell type proportions coming from epigenetic profiles makes it possible to consider cell type specificity in epigenetic analyses. These analyses are important for two reasons. First, cell types could be a confounding factor in EWAS^23^. Previous EWAS in the brain have mainly dealt with potential confounding by cell type by correcting for two different brain cell type populations: NeuN+ (neuronal) and NeuN− (non-neuronal) cells based on previously described deconvolution panels^24^. A more recent brain cell type reference panel allows for deconvolution to estimate up to seven different brain cell types: endothelial and stromal cells, astrocytes, microglial cells, oligodendrocytes, and GABA- and glutamatergic neurons^25^. Accounting statistical analyses for a more granular panel of cell types might reduce the confounding of results introduced by cell type proportions. Thus, it would be possible to determine whether previous DNA methylation-ADHD associations observed in the ACC and CN^12^ could be ascribed to brain region-specific DNA methylation profiles and their distinct roles in ADHD aetiology rather than reflecting cell type proportion differences. Secondly, a more granular cell type overview can determine which brain cell types are driving the DNA methylation associations observed in the bulk tissue analyses, thereby making it possible to interpret DNA methylation associations in a more biologically meaningful way. As previous studies have hinted towards specific cell types playing potential roles in ADHD^20,26^, cell type-specific EWAS could show which DNA methylation associations are associated with ADHD in a cell type-specific manner.

In this study we aimed to 1) investigate whether there was a difference in seven distinct brain cell type proportions (endothelial cells, stromal cells, astrocytes, microglia, oligodendrocytes, and GABA- and glutamatergic neurons) in the ACC and CN between those with and without ADHD; 2) to examine diagnostic differences in DNA methylation correcting for these seven distinct brain cell type proportions, contrasting against results when an adjustment is made solely for neuronal versus non-neuronal cells (NeuN+/NeuN−), and 3) to perform brain cell type-specific differential DNA methylation analysis.

## Subjects and methods

### Postmortem brain tissue

In total, 58 postmortem brain samples from both the anterior cingulate cortex (ACC) and caudate nucleus (CN) were used, collected from the National Institute of Mental Health Human Brain Collection Core (HBCC; ten cases, fourteen controls), Brain Tissue Donation Program at the University of Pittsburgh (five cases, five controls), and the University of Maryland Brain and Tissue Bank (ten cases, fourteen controls). Procedures at the NIMH HBCC procedures are approved by an oversight committee and the NIH Department of Bioethics and all study sites acquired postmortem tissue and conducted all related procedures under protocols approved by their local IRB. All methods were carried out in accordance with guidelines and regulations from the respective institutes. Informed consent was obtained by next of kin. ADHD diagnosis was determined by interviewing next of kin using DSM-IV criteria in combination with reviewing prior records. Exclusion criteria included the presence of major neurological disorders or schizophrenia, but not developmental disorders such as autism spectrum disorder. Controls were defined as individuals with no history of mental illness. In the remainder of the manuscript, we refer to the controls as unaffected comparison individuals. Mean age of individuals was 21 and 23 years old, and predominantly male (79%). In total, 55% of individuals were White. A complete description of the brain samples is given in the supplement.

### DNA methylation

Tissue for DNA methylation analyses was available from the ACC from 55 donors and the CN from 58 donors. All subsequent lab protocols were performed centralized on all the samples at the same time. DNA was extracted from the bulk tissue, and bisulfite converted (EZ DNA Methylation kit, Zymo Research, Tustin, CA, USA), and the epigenotyping was performed by the Genomics Core of the NHGRI using the HumanMethylationEPIC BeadChip (Illumina, San Diego, CA, USA), ensuring that samples from different study centers were randomized over plates. Preprocessing of the DNA methylation was done using the Psychiatric Genomics Consortium (PGC) ADHD Working Group pipeline for EWAS. More details on DNA methylation preprocessing is given in the supplement.

### Brain cell type proportions

Brain cell type proportions were estimated based on epigenomic deconvolution, which allows to estimate cell type proportions in bulk tissue. One way to estimate cell type proportions through epigenomic deconvolution is by making use of reference panels. These reference panels contain selected DNA methylation sites which have the most discriminative power of a predefined set of cell types, based on external samples. Multiple different reference panels for a multitude of tissues exist, and even within tissues, multiple reference panels reflecting different cell type populations exist. In this study, we made use of two different reference panels: First, NeuN+and NeuN− cell type proportions were estimated^24^. Secondly, seven different brain cell type proportions (endothelial cells, stromal cells, astrocytes, microglia, oligodendrocytes, GABA- and glutamatergic neurons) were estimated with the *HiBED* (Hierarchical Brain Extended Deconvolution) R package^25^. Difference in estimated cell type proportions in the brain between individuals with ADHD and unaffected comparison individuals was assessed by correcting for sex, age, substance use, and whether the individual had any comorbidities. The variable ‘comorbidities’ was coded as a binary variable for whether the donor had any comorbidities (specifically: bipolar affective disorder, autism spectrum disorder, major depressive disorder, impulse control disorder, adjustment disorder, or dysthymia, Supplementary Table 1).

### Epigenome-wide association studies

Epigenome-wide association studies (EWAS) were conducted using *cpg.assoc()* function from the *minfi* R package^27^. The analyses were conducted separately for ACC and CN. Covariates in the EWAS models were selected based on an approach previously employed by others and our group^12^. We extracted principal components (PCs) based on the DNA methylation data and retained the components with eigenvalues above one. For the ACC, the first ten PCs were retained, accounting for 46% of variance, and for the CN, the first fourteen PCs accounted for 49% of variance. Then, we tested for associations between these PCs and relevant covariates with Spearman correlations (continuous covariates) or a Kruskal–Wallis test (categorical covariates). The covariates considered here were age of death, gender, comorbidities, substance abuse, mode of death, clinical evidence level, the first five ancestry components, processing batch, brain bank of origin, postmortem interval, the first ten PCs derived from control probes, and a DNA methylation-based smoking score. Covariates associated with any PC at a Bonferroni corrected *p*-value < 0.05 were added to the statistical model. Finally, we also included variables associated with diagnosis at a Bonferroni corrected *p*-value < 0.05. The final model for the ACC included age at death, substance use, one technical component and seven cell type proportions. For the CN, age at death, smoking score, substance use, two ancestral components, two technical components and seven different brain cell type proportions were added to the model. A complete overview on the building of the statistical models can be found elsewhere^12^.

Cell type-specific DNA methylation associations were calculated with the *CellDMC()* function from the *EpiDISH* R package through incorporation of a statistical interaction term of the cell type and the phenotype of interest (cell type 1–7 * ADHD status). The underlying assumption here is that if a DNA methylation pattern is specific to one cell type, the differential methylation should be most prominent when the analysis is restricted to samples with the highest proportion of that cell type^28^. We extracted coefficients for the interaction between cell type and ADHD status, as well as the main coefficient for each cell type. For each cell type, the same covariates were included as in the original bulk analyses. False-discovery rate (FDR) was used to adjust *p*-values for multiple testing. An FDR q < 0.05 was considered to be statistically significant.

### Differentially methylated regions

To identify subtle but consistent differentially methylated regions (DMRs) associated with ADHD diagnosis, we employed mCSEA based on Gene-Set Enrichment Analysis (GSEA)^29,30^. The analyses were performed for DNA methylation sites classified as genes (annotated to gene body), promoters (annotated to TSS1500, TSS200, 5’UTR, or 1st Exon), or CpG islands (CGI; annotated to Islands, N-shore, S-shore, N-shelf, or S-shelf) according to the Illumina b2 manifest file. Only regions with five or more probes were analysed. The DMRs were mapped to genes using the leading edge CpG probes that contribute most to its differential methylation. See supplementary methods for further details. If the gene functionality involved any of the words “serot”, ‘gaba”, “dopa”, “glut”, or “neur”, it was flagged as a gene with a neural-related function. False-discovery rate (FDR) was used to adjust *p*-values for multiple testing. An FDR q < 0.05 was considered to be statistically significant.

### Downstream enrichment analyses

Gene Ontology (GO) term enrichment was performed using *gometh()* in the *missmethyl* R package, which takes probe distribution biases into account^31^. We included the leading DNA methylation sites indicated in the mCSEA analysis with an FDR < 0.05. Next, we tested whether the DMRs associated with ADHD were enriched for genetic risk variants for psychiatric disorders, including ADHD, based on single nucleotide polymorphisms (SNPs) identified by previously published genome-wide association studies (GWAS). We used Multi-marker Analysis of GenoMic Annotation (MAGMA)^32^ to test whether SNP associations implied by GWAS reflected differential methylation. The GWAS used as input were for ADHD^16,33^, autism spectrum disorder^34,35^, major depressive disorder^36,37^, bipolar affective disorder^38,39^, schizophrenia^40,41^, Tourette Syndrome^42^, obsessive compulsive disorder^43^, Alzheimer’s disease^44,45^, alcohol use disorder^46^, and rheumatoid arthritis^47^ (the latter as negative control). Analyses were done on all DMRs, and on DMRs in genes, promoters, and CpG islands (CGI). False-discovery rates (FDRs) were calculated for each individual analysis to adjust *p*-values for multiple testing. An FDR q < 0.05 was considered to be statistically significant.

## Results

### No differences in cell type proportions between individuals with ADHD and unaffected comparison individuals in either brain region

Proportions of seven different brain cell types (endothelial cells, stromal cells, astrocytes, microglia, oligodendrocytes, GABA- and GLU neurons) were estimated based on epigenomic deconvolution. The ACC and CN displayed differences in brain cell type proportions (*p* < 0.001, Supplementary Fig. 1). Generally, the ACC showed more variability between individuals than the CN (e.g., 25–65% vs. 40–60% oligodendrocytes; 5–30% versus 5–15% glutamatergic neurons; *p* < 0.001, Supplementary Fig. 1). We then compared proportions and distributions of the estimated cell type proportions between individuals with ADHD and unaffected comparison individuals (the latter reflecting individuals without a history of mental illness, including ADHD). None of the estimated brain cell type proportions or their distributions were different between individuals with ADHD and unaffected comparison individuals in either the ACC or CN after correcting for sex, age, and comorbidities (*p* < 0.05).

### The anterior cingulate cortex and caudate nucleus showed different DNA methylation associations with ADHD

**Fig. 1:**
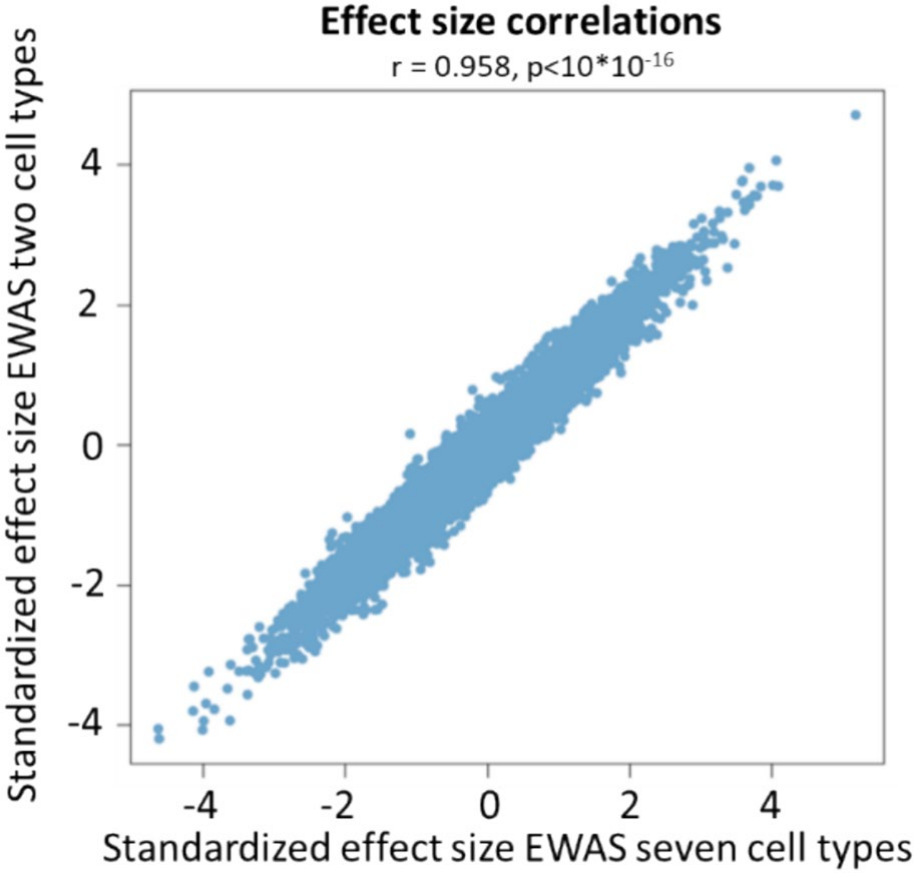
Effect sizes correlated between EWAS corrected for two and seven cell types. EWAS on ADHD was performed in the anterior cingulate cortex (ACC) using statistical models correcting for both two different cell populations (NeuN+/NeuN−) and seven different cell types (endothelial cells, stromal cells, astrocytes, microglia, oligodendrocytes, GABA- and GLU neurons). Standardized effect sizes (effect size – mean effect size / standard deviation) of all probes tested are plotted for both models (x-axis = seven cell types, y-axis = two cell types). Pearson’s correlation coefficient is depicted at the top of the graph.

An EWAS for ADHD was performed on bulk tissue from both the ACC and CN (Supplementary Table 7, Supplementary Table 15), contrasting the findings for adjustment for seven cell types against the findings when making a more standard adjustment for neuronal versus non-neuronal (NeuN+/NeuN−) cells. Neither EWAS model showed test-statistic inflation (ACC lambda = 1.086, CN lambda = 1.028; Supplementary Fig. 2). In both set of analyses, no single DNA methylation site was statistically significantly associated with ADHD status. The standardized effect sizes for both statistical models, i.e., with two and seven different cell types, in the ACC were highly correlated and comparable in their magnitude (Pearson’s r = 0.958; Fig. 1). Similar findings were observed for the CN. We ranked the epigenetic associations with ADHD diagnosis in the ACC and CN based on *p*-values, and extracted the top ten most significant associations of both the statistical model adjusted for two cell types and the statistical model adjusted for seven cell types. We found that the ranking of the single CpG sites changed considerable for many single probes (Table 1).

**Table 1:** Most significant DNA methylation-associations with ADHD across two brain regions and across two statistical models.

NeuN+/NeuN−						Seven cell types					
ACC			Caudate			ACC			Caudate		
#CpG site	CpG	Rank in Seven cell type analysis	#CpG site	CpG	Rank in Seven cell type analysis	#CpG site	CpG	Rank in two cell type analysis	#CpG site	CpG	Rank in two cell type analysis
1	cg15416329	32	1	cg21494722	29	1	cg16357179	295	1	cg12889533	8
2	cg20426042	43	2	cg10362977	16	2	cg12806067	5	2	cg26519659	70
3	cg25054793	17	3	cg14997559	71	3	cg27286106	43	3	cg25111130	10
4	cg27599079	8	4	cg26293202	6	4	cg18485154	10	4	cg08618909	63
5	cg12806067	2	5	cg13544743	141	5	cg07743843	20	5	cg12952561	3929
6	cg17737641	29	6	cg05863023	133	6	cg27305917	112	6	cg26293202	4
7	cg00588691	55	7	cg23253569	11	7	cg02985381	13	7	cg02941596	90
8	cg13153394	20	8	cg12889533	1	8	cg27599079	4	8	cg05955013	286
9	cg13689470	25	9	cg25752955	477	9	cg24888975	12	9	cg04021856	27
10	cg18485154	4	10	cg25111130	3	10	cg00183742	45	10	cg08577238	51

CpG sites are ranked (CpG site #) based on their significance in the bulk analyses corrected with two different type of reference panels: NeuN+/NeuN− (left side of the table) or seven cell types (endothelial cells, stromal cells, astrocytes, microglia, oligodendrocytes, GABA- and GLU neurons, right side of the table) for both the anterior cingulate cortex (ACC) or the caudate nucleus (Caudate). Top ten CpG sites are shown for each analysis and the respective rank in the analysis with the different cell type reference panel is given as comparison*.*

### Changes in DMR analyses on EWAS adjusted for two versus seven cell types

There was considerable overlap in the DMRs that were implicated through the EWAS that adjusted for seven (Supplementary Table 2) and those that adjusted for two cell types (overlap of 283 DMR (58.5%) for the ACC, and 84 (54.5%) in the CN). However, compared to correcting for two cell types, correcting for seven cell types yielded fewer DMRs, and also fewer DMRs annotated to genes with a neural-related function (13.4%—> 11.6% in the ACC, *p* = 0.36; 20.4%—> 11% in the CN, *p* = 0.011).

### ADHD was associated with cell type-specific DNA methylation profiles, predominantly in the caudate nucleus

Cell type-specific DNA methylation-ADHD associations were calculated through the incorporation of a statistical interaction term of the different cell types and ADHD case–control status (Supplementary Table 3, Supplementary Tables 7–22). There were three significant DNA methylation-ADHD associations in microglia in the CN (lambda = 0.997; Table 2). These DNA methylation sites were annotated to genes involved in lipid metabolism (Diacylglycerol O-acyltransferase 1; *DGAT1*), a glutamate receptor (Glutamate ionotropic receptor delta type subunit 1; *GRID1*), and a gene involved in receptor recycling (TBC1 Domain Family Member 16; *TBC1D16*).

**Table 2:** Overview of statistically significant cell type-specific DNA methylation-ADHD associations in the anterior cingulate cortex and caudate nucleus.

	CpG site	*p*-value	FDR	Effect size	Standard error	Gene
CN						
Microglia	cg07086847	9.97*10^–10^	− 1.11*10^–2^	8.09*10^–4^	1.26*10^–3^	*DGAT1*
	cg15547344	1.22*10^–7^	2.62*10^–2^	3.87*10^–2^	3.93*10^–3^	*GRID1*
	cg00508479	1.43*10^–7^	4.02*10^–2^	3.87*10^–2^	6.09*10^–3^	*TBC1D16*
Stromal cells	cg24770798	6.58*10^–9^	5.34*10^–3^	0.0970	0.0127	
ACC						
Stromal cells	cg03314803	3.68*10^–9^	3.02*10^–3^	− 1.00	0.179	*FAM19A5*
	cg02305803	1.10*10^–8^	3.09*10^–3^	− 1.00	0.190	*RBCK1*
	cg14538625	1.13*10^–8^	3.09*10^–3^	− 1.00	0.201	*ARID1B*
	cg01208296	2.13*10^–8^	4.37*10^–3^	− 1.00	0.179	*IP6K3*
	cg10118435	5.29*10^–8^	8.04*10^–3^	− 0.580	0.0829	*CTNND1*
	cg14525390	5.88*10^–8^	8.04*10^–3^	− 1.00	0.199	*HECW1*
	cg07918589	8.12*10^–8^	9.52*10^–3^	− 1.00	0.185	
	cg01213645	3.24*10^–7^	3.03*10^–3^	− 1.00	0.202	*SHANK2*
	cg20369554	3.32*10^–7^	3.03*10^–3^	− 1.00	0.230	
	cg03187338	4.11*10^–7^	3.37*10^–2^	− 0.999	0.157	
	cg03072681	5.67*10^–7^	4.23*10^–2^	0.505	0.0817	
	cg09106629	6.99*10^–7^	4.78*10^–2^	− 0.639	0.105	*TTC7B*

ACC, Anterior cingulate cortex; CN, Caudate nucleus.

FDR, False Discovery Rate.

There were twelve single DNA methylation sites showing an epigenome-wide association with ADHD status for stromal cells in the ACC (lambda = 1.092) compared to one stromal association in the NC (lambda = 0.967). However, given the low proportion levels of stromal cells (Supplementary Fig. 1), leading to effect sizes of − 1.00 to 1.00, these findings most likely reflect a statistical shortcoming in cell types with low abundance rather than a biological effect. Therefore, these findings should be interpreted with caution.

Moving from cell type-specific single DNA methylation sites to cell type-specific DMRs, we identified more DMRs in the ACC compared to the CN. In the ACC, glutamatergic neurons showed the largest number of DMRs (1122 regions). In the CN, astrocytes showed the largest number of DMRs (867 regions) (Supplementary Table 4).

We compared the EWAS results in bulk tissue with the results from the cell type-specific analyses. Such analyses point towards the brain cell types that may be driving the EWAS findings in bulk tissue analyses. In the ACC, DMR patterns in bulk tissue overlapped mostly with the astrocyte EWAS (32.1%) and GABAergic neuron EWAS (31.2%), and these overlaps were both higher than expected by chance (*p*s < 2.2*10–16). For the NC, DMR patterns in bulk tissue were most similar to that of microglia (21.5%), oligodendrocytes (23.4%) and glutamatergic neurons (23.8%), all showing overlap higher than expected by change (*p*s < 2.2*10^–16^).

In the ACC, 320 DMRs overlapped for all seven cell types, of which 247 were also identified in bulk ACC tissue. For the CN, 74 DMRs were overlapping between all cell types, of which 47 were also present in the bulk analysis, including *LHX3*, *PRDM16*, and *BAIAP2* (Supplementary Table 4).

Gene Ontology (GO) term enrichment revealed stronger associations with ADHD diagnosis in the CN than the ACC for all analyses (bulk corrected for two or seven cell types, endothelial cells, stromal cells, astrocytes, and GABAergic and glutamatergic neurons), except for microglial cells and oligodendrocytes. This is in contrast with the earlier findings of the amount of DMRs associated with ADHD in the ACC compared to the CN when adjustment was made for either two or seven cell types. The most GO term enrichments were identified for GABAergic neurons in the CN (Supplementary Table 5).

### Enrichment of genetic risk variants for ADHD was driven by endothelial and stromal cells, microglia and glutamatergic neurons

We tested whether the genes implicated by DMRs were enriched for genes associated with different psychiatric disorders based on genome-wide association studies (Table 3, Supplementary Table 6). In the ACC, genetic risk variants for ADHD were not enriched in the DMRs identified in either bulk tissue or any cell type. In the CN, however, DMRs in bulk tissue were enriched for genetic variants for ADHD when corrected for two cell types, but not when corrected for seven distinct cell types. The cell type-specific analyses revealed that the genetic enrichment for ADHD in the CN was driven by endothelial and stromal cells, as well as microglia and GABAergic neurons. Similarly, genetic enrichment for ASD in the ACC was mainly driven by endothelial and stromal cells, microglia and glutamatergic neurons. Lastly, genetic enrichment for bipolar disorder was absent in any of the DMR analyses in the ACC, but present in the CN, mainly driven by endothelial and stromal cells, as well as astrocytes.

**Table 3:** Enrichment for genetic risk variants of psychiatric disorders in differentially methylated regions associated with ADHD status.

	Bulk 2 cell types	Bulk 7 cell types	Endothelial	Stromal	Astrocytes	Microglial	Oligodendrocytes	GABA	GLU
ACC									
ADHD									
AD									
ASD	**All**		**All; genes**	**All; genes**		**CGI**			**All; promoters**
AUD									
BD									
MDD		**CGI**				**Genes**		**Genes**	
OCD									
SCZ									
TS									
Arthritis									
Caudate									
ADHD	**All; genes**		**All; genes; promoters**	Genes*	All*	**Genes**		**All; genes**	
AD									
ASD	**All; genes**								
AUD									
BD	**Genes**		**Genes**	**Genes**	**Genes**				
MDD									
OCD	**All**			**All; genes**					
SCZ									
TS									
Arthritis									

Analyses were performed on all DMRs (All), DMRs in genes (genes), DMRs in promoters (promoters), and DMRs in CpG island (CGI). The enrichments shown in bold are the enrichment analyses with a *p*-value < 0.05. An * indicates that the *p*-values of the enrichment are lower than 6*10^–4^, corrected for the number of tests performed. A complete overview of all enrichments can be found in Supplementary Table 6.

ACC, Anterior cingulate cortex; Caudate, Caudate nucleus; ADHD, Attention-deficit/hyperactivity disorder; AD, Alzheimer’s disease; ASD, Autism spectrum disorder; AUD, Alcohol use disorder; BD, Bipolar disorder; MDD, Major depressive disorder; OCD, Obsessive–compulsive disorder; SCZ, Schizophrenia; TS, Tourette’s Syndrome.

## Discussion

Using DNA methylation data from bulk brain tissue, we find fewer associations with ADHD when adjustment is made for seven distinct brain cell types, as opposed to two different brain cell type classes. However, diagnostic DNA methylation signals were prominent in cell type-specific analyses, particularly for microglia and GABAergic neurons. These cell type-specific signals both implicated genes involved in brain development, and overlapped with genes enriched for common variant risk for ADHD.

### No differences in brain cell type proportions between those with and without ADHD

There were no differences in brain cell type proportions in either the ACC or the CN in individuals with ADHD compared to unaffected individuals. Even though functional and structural differences in these brain regions have been observed in association with ADHD^13–15^, our findings suggest these observations are most likely not due to differences in cell type proportions.

### Diagnostic differences in DNA methylation when adjusting for different brain cell type reference panels

Our findings speak to the importance of considering the appropriate form of adjustment for cell types –(NeuN+/NeuN−^24^ or seven cell types^48^). There was overall a high correlation in the effect sizes of single DNA methylations sites when correcting for two or seven different cell types, which is perhaps reassuring, but the top probe ranking differed between the analyses. It should be noted that the ranking of single probes can change due to random noise; however, the other downstream analyses, including DMRs, GO term enrichment, and genetic risk variant enrichment, also showed differences between analyses corrected for different cell type reference panels. Therefore, the choice of reference panel used is dependent on the context of the research question at hand. We recommend the three following considerations: 1) If the study aim is to perform an exploratory hypothesis-generating analysis, correcting for two cell types (NeuN+/NeuN−) suffices. Results will likely inform on the relevant cellular mechanisms involved, as cellular heterogeneity is not completely account for, illustrated by the fact that DMR and GO term enrichment pointed towards findings related to specific cell types (e.g., “oligodendrocyte differentiation”) when corrected for two cell types. After correcting for seven different cell types, only the findings that are on average different in *all* brain cell types between ‘cases’ and ‘controls’ remain. 2) Especially when sample sizes are relatively small, correcting for two cell types could be beneficial: adding more cell types as covariates to the statistical EWAS model will result in fewer degrees of freedom, and consequently, less power to detect statistically significant results. 3) If the study aim is to unravel cellular processes underlying the phenotype to be studied, cell type-specific analysis, which relies on the extended reference panel with seven cell types, reveals the most biologically relevant information. Results aid in elucidating the distinct processes associated with the brain phenotype of interest for each of the seven cell types.

### Cell type-specific differential DNA methylation between individuals with ADHD and unaffected comparison individuals

Consistent with previous studies showing larger gene expression differences between individuals with ADHD and unaffected comparison individuals in the CN than in the ACC, our study detected more DNA methylation associations in the CN than in ACC in all statistical models (i.e., corrected for two cell types and seven cell types, as well as the cell type-specific models). On the cell type-specific level, most DMRs and GO term enrichments in the ACC were observed for glutamatergic neurons, and genetic risk factors for ADHD were mainly enriched in the DMRs of these glutamatergic neurons and microglial cells. Similarly, in the CN, microglial cells showed differential DNA methylation profiles on the single site level. However, on the DMR level, most DNA methylation associations were observed in astrocytes. On top of that, most GO term enrichments and genetic risk enrichment for ADHD was observed in GABAergic neurons. Thus, in the ACC and CN, excitatory and inhibitory neurons were associated with ADHD diagnosis, respectively. A meta-analysis of 25 previous proton magnetic resonance spectroscopy studies has shown that children with ADHD had higher levels of glutamate and glutamine in the right medial frontal area specifically compared to children without ADHD^49^. However, several individual studies are of particular relevance as they focus on changes in glutamate in our regions of interest: one study has shown that glutamate-to-creatine rations in the ACC were marginally lower in individuals with ADHD compared to individuals without ADHD during a cognitive control task^50^. A second study showed higher levels of glutamate in the ACC in individuals with ADHD compared to individuals without ADHD. These levels of glutamate were positively correlated with hyperactivity and impulsivity symptoms^51^. On the other hand, children with ADHD had lower GABA-to-creatine levels in the striatum (including the CN), but not in the ACC^52^. These findings support ours that glutamatergic neurons in the ACC and GABAergic neurons in the CN might mainly be involved in the development and/or persistence of ADHD.

The cell type-specific EWAS pointed to a potential role for astrocytes and microglia, which form part of the brain’s complex immune system. This corresponds to the previous evidence of a possible link between the immune system and ADHD^20,53–55^. Furthermore, genetic and epigenetic risk factors for ADHD have been found enriched in astrocyte cells of the mouse brain^18^ and human peripheral tissues^20^. On the single-site level, one of the three statistically significant DNA methylation sites in microglia in the CN is involved in lipid metabolism (*DGAT1*). More specifically, *DGAT1* catalyses the final step in triglyceride biosynthesis. Triglycerides are transported by lipoproteins such as VLDL^56^. Based on GWAS results, ADHD is genetically correlated with triglyceride levels (rg = 0.18), High-Density Lipoprotein (HDL) cholesterol (rg = − 0.21), and medium Very Low-Density Lipoprotein particles (rg = 0.24)^16^. Triglycerides can cross the blood–brain barrier (BBB) and directly affect brain function^57^. However, microglia also express *DGAT1*^58,59^, and the production of triglycerides is essential for microglia to properly respond to extrinsic immune activation^60^. Disturbances in microglial lipid metabolism can lead to neuroinflammation and disease^61^ and might be an underlying risk factor for ADHD^55^.

### Strenghts and limitations

There are several important limitations to the current study. First, postmortem brains were collected at multiple study sites and could have introduced batch effects in our analyses. However, by using common tissue preparation pipelines, as well as DNA methylation acquisition and quality control pipelines, heterogeneity introduced by the study site was limited. Second, the sample size in the current study was modest, and an increase in sample size could lead to a better-powered study to identify more cell type- and DNA methylation patterns associated with ADHD, especially for analyses relying on interaction terms. Relatedly, it should also be noted that some estimated cell type proportions are very low, e.g., stromal proportions. Performing statistical analyses with interaction terms with cell type proportions of low abundance may lead to overestimation of effect sizes. Third, in the current analysis, individuals with ADHD and comorbidities were included, which could have potentially influenced the outcome of our analysis given the overlap in cognitive and emotional features of these diagnoses. Directions for future research include analyses on individuals with ADHD and without comorbidities to isolate the ADHD-specific DNA methylation associations. Fourth, two different brain regions were investigated in the current study, and while chosen based on previous knowledge^13–15^, other brain regions could also be relevant in the aetiology of ADHD. Fifth, the cell type proportions used were derived from estimations based on epigenomic deconvolution approaches and not based on cell count data. However, the use of such reference panels also has its benefits in epigenetic research as the data can easily be updated when the research field is progressing. Sixth, even though we were able to identify genetic enrichments for psychiatric disorders in the DMRs identified in specific cell types, we were not able to test whether genetic risk factors for psychiatric disorders alter DNA methylation profiles associated with ADHD diagnosis. To reveal a more mechanistic link between GWAS loci and DNA methylation levels, a methylation quantitative trait loci (mQTL) analysis is needed. The lack of mQTL data for the ACC and CN makes it impossible to perform such analyses, but would be a key direction of future research of DNA methylation in the context of ADHD. Lastly, we were not able to perform any type of replication in an independent dataset, as this is – to our knowledge – the only cohort of brain DNA methylation profiles of individuals with ADHD available to the research community.

## Conclusions

In the current study, we showed that cell type-specific epigenetic patterns in two distinct brain regions in the cortico-striatal network were differentially associated with ADHD diagnosis. Specifically, DNA methylation differences in the ACC were mainly driven by glutamatergic neurons, whereas differences in the CN were mainly driven by GABAergic neurons. Furthermore, the role for microglia in both brain regions suggests a possible role for neuroinflammation, mediated by atypical lipid metabolism, as a potential contributory mechanism. Lastly, we showed that the results of EWAS were dependent on the cell type reference panels used in statistical analyses, and which ones to use is dependent on the research question to be answered. In summary, our findings offer new hypotheses for understanding the molecular mechanisms underlying ADHD and highlight important considerations for conducting EWAS analyses in brain tissue.

## Supplementary Information

Supplementary Information 1.

Supplementary Information 2.

Supplementary Information 3.

Supplementary Information 4.

Supplementary Information 5.

Supplementary Information 6.

Supplementary Information 7.

Supplementary Information 8.

Supplementary Information 9.

Supplementary Information 10.

Supplementary Information 11.

Supplementary Information 12.

Supplementary Information 13.

Supplementary Information 14.

Supplementary Information 15.

Supplementary Information 16.

Supplementary Information 17.

Supplementary Information 18.

Supplementary Information 19.

Supplementary Information 20.

Supplementary Information 21.

## Data Availability

Data are being deposited in NIMH Data Archive under Collection 3151, experiment 2443 ([https://nda.nih.gov/edit/_collection?id=3151)](https://nda.nih.gov/edit_collection.html?id=3151).
