# Supplementary material for: Brain region and cell type-specific DNA methylation profiles in association with ADHD
Source: Sci Rep. 2025 Oct 8;15:35078. doi: 10.1038/s41598-025-18724-1 (PMC12508121; doi:10.1038/s41598-025-18724-1)
Supplement: Supplementary file 8 — Supplementary Information 8. [file 41598_2025_18724_MOESM8_ESM.docx]

**Brain region and cell type-specific DNA methylation profiles in association with ADHD**

Mandy Meijer, Gustavo Sudre, Kwangmi Ahn, Maggie Po Yuan Fu, Philip Shaw

**Supplementary material**

**Supplementary methods**

*Postmortem brain tissue*

ADHD diagnoses were assessed at every study site by clinician interviews based on the DSM with the next of kin. For the NIMH HBCC and Maryland Brain Collection, postmortem interviews were performed with at least one family member of the deceased by a trained psychiatrist, social worker, or other health care professional using the Structured Clinical Interview for DSM-IV-clinician version^1^ along with psychiatric record reviews using the Diagnostic Evaluation after Death.^2^ Where available other pertinent records such as Medical Examiner’s Office or medical records were summarized and reviewed by two psychiatrists who arrived at a consensus for DSM diagnoses.^3^ The University of Pittsburgh Brain Tissue Donation Program similarly conducted a postmortem interview with the family of the deceased using a structured interview for the DSM 5. Interview data were reviewed by a team of psychiatrists and psychologists along with medical records and publicly available records to arrive at a DSM diagnosis.^4^

Brain tissue was sectioned as coronal slabs at autopsy and then frozen at –80 °C. Dissections were performed on frozen tissue held on dry ice in small batches for about 30 minutes. Dissections targeted the dorsal anterior cingulate cortex (ACC), above the genu of the corpus callosum, and the head of the caudate (Caudate Nucleus; CN).

*DNA methylation*

DNA methylation was processed by importing raw IDAT files into R using the *minfi* package.^5^ Samples were excluded if the median unmethylation or methylated signal was lower than 13, sodium bisulfite conversion rates were <80%, detection p-values>0.01 in more than 1% of probes, the overall methylation call rate<95%, or reported sex did not match predicted sex using *minfi::getSex().* Principal components (PCs) calculated across all probes were used to identify outliers, and any sample with more than 2 standard deviations from the mean for both PC1 and PC2 were removed. Then probes were removed if the detection p-value>0.01 in more than 2% of samples or when the probes were located on the sex chromosomes, known to cross-hybridize, not measuring DNA methylation in CpG sites, and probes that contain common SNPs. Filtered probes were quantile normalized using the CPACOR pipeline.^6^ In total, 811,639 probes were retained for the CN, and 820,051 probes were retained for the ACC. Two CN samples were removed based on median intensity plots, and four ACC samples were removed as outliers based on PCs performed in SNPs identified from the DNA methylation array. The final postmortem brain EWAS thus included data from 51 ACC and 56 caudate specimens.

A smoking score was calculated based on the cleaned DNA methylation data.^7^

*Differentially methylated regions*

We performed mCSEA based on Gene-Set Enrichment Analysis, ranking all CpG sites based on the t-statstics derived from the association between DNA methylation profiles and ADHD diagnosis state. An enrichment analysis is performed on CpG sites in pre-defined regions using GSEA, implemented in *fgsea.* Regions with CpG sites over-represented in the ordered list of CpG sites are defined as differentially methylated regions (DMRs). The DMR analysis was conducted separately for probes in gene bodies, promoter regions, and CpG islands, and only regions with five or more probes were considered. For the ACC, this resulted in 15,155 regions for gene bodies, 19,162 for promoters, and 24,657 for CpG islands. For the CN, this resulted in 15,076 regions for gene bodies, 19,073 for promoters, and 24,626 for CpG islands. The leading probes of a DMR were mapped to the nearest gene using the R package IlluminaHumanMethylationEPICanno.ilm10b2.hg19, (within 1500 bp upstream or downstream).

**Supplementary results**


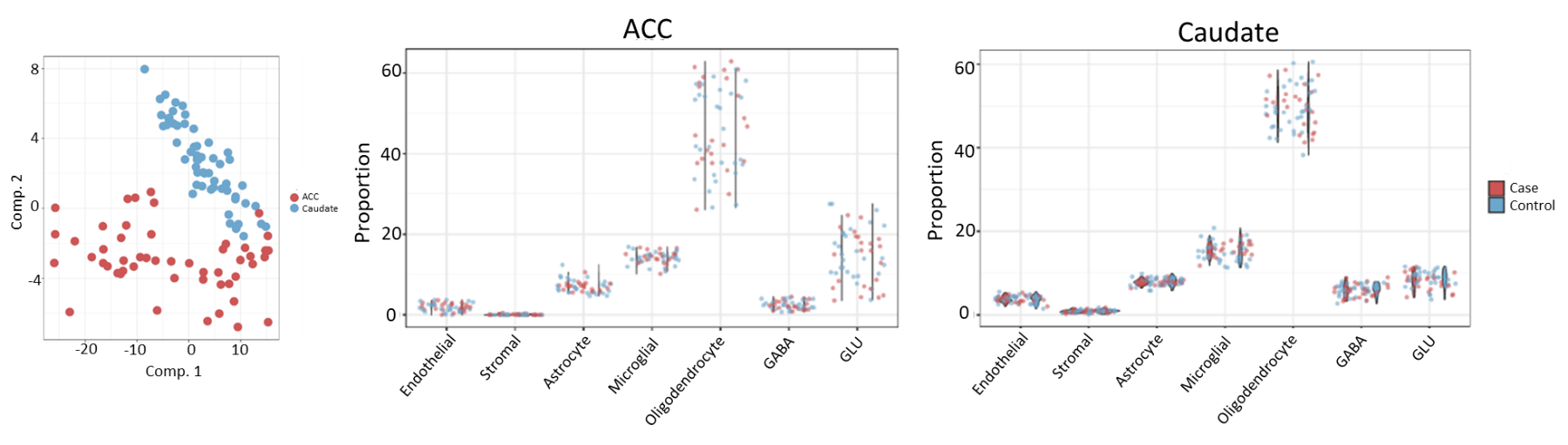
**Supplementary Figure 1 Estimated cell type proportions in the anterior cingulate cortex and caudate nucleus do not differ between individuals with ADHD and unaffected comparison individuals.** Brain cell type proportions were estimated with the HiBED epigenomic deconvolution panel. Principal component analyses showed that the anterior cingulate cortex (ACC; red) showed different cell type proportion patterns than the caudate nucleus (blue) (left panel). There were no differences in brain cell type proportions between individuals with ADHD (red) compared to unaffected individuals (blue) in either the ACC (middle panel) or caudate nucleus (right panel).


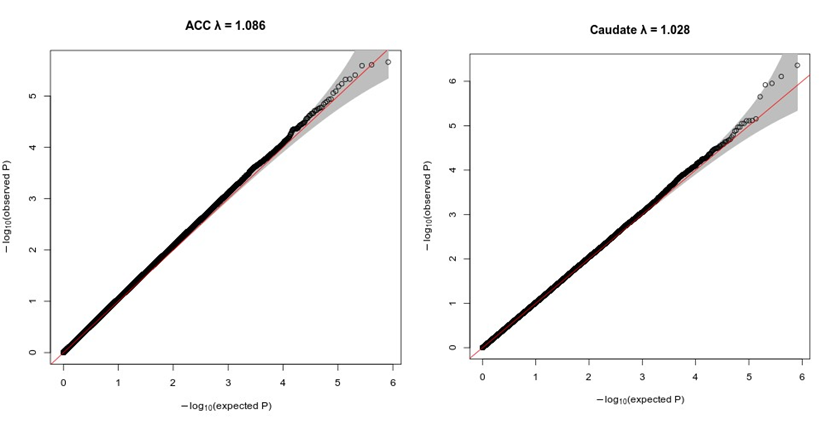
**Supplementary Figure 2 Quantile-quantile plots for epigenome-wide association studies in the anterior cingulate cortex and caudate nucleus bulk tissue did not show test-statistic inflation.** The x-axis shows the expected -log10(p-value) and the y-axis shows the observed -log10(p-value). The inflation factor (λ) is given at the top of each plot. ACC = anterior cingulate cortex; Caudate = Caudate nucleus.

**Supplementary Table 1 Comorbidities in individuals with ADHD**

| **Comorbidities** | **N** |
| --- | --- |
| None | 16 |
| ASD | 1 |
| MDD | 5 |
| BPAD_NOS; Impulse control disorder | 1 |
| **Total** | **23** |

**Supplementary Table 2 Differentially methylated regions in bulk epigenome-wide association study for ADHD in both the anterior cingulate cortex and caudate nucleus corrected for seven different brain cell types**

*See Excel “Supplementary Table 2”*

**Supplementary Table 3 Overview of statistically significant associations in cell type-specific epigenome-wide association study for ADHD in both the anterior cingulate cortex and caudate nucleus**

|  | ACC | | CN | |
| --- | --- | --- | --- | --- |
| Cell type | # Significant associations | Lowest adjusted p-value | # Significant associations | Lowest adjusted p-value |
| Endothelial cells | 0 | 0.126 | 0 | 0.0962 |
| **Stromal cells** | **12** | **0.003** | **1** | **0.00534** |
| Astrocytes | 0 | 0.09 | 0 | 0.999 |
| **Microglia** | 0 | 0.999 | **3** | **0.000809** |
| Oligodendrocytes | 0 | 0.999 | 0 | 0.479 |
| GABA | 0 | 0.347 | 0 | 0.751 |
| GLU | 0 | 0.999 | 0 | 0.999 |

ACC = Anterior cingulate cortex; CN= Caudate nucleus

Bold text indicates cell types with statistically significant associations with ADHD case-control status.

**Supplementary Table 4 Overview of differentially methylated regions of cell type-specific epigenome-wide association study for ADHD in the anterior cingulate cortex and caudate nucleus**

*See Excel “Supplementary Table 4”*

**Supplementary Table 5 Gene Ontology term enrichment of bulk and cell type-specific differentially methylated regions in ADHD in both the anterior cingulate cortex and caudate nucleus**

*See Excel “Supplementary Table 5”*

**Supplementary Table 6 Enrichment of genetic risk variants for psychiatric disorders in cell type-specific differentially methylated regions in ADHD in both the anterior cingulate cortex and caudate nucleus**

*See Excel “Supplementary Table 6”*

**Supplementary Table 7-14 Summary statistics of epigenome-wide association study for bulk data, and seven different cell types**

*See text files “Supplementary Table 7-14_ACC”*

**Supplementary Table 15-22 Summary statistics of epigenome-wide association study for bulk data, and seven different cell types**

*See text files “Supplementary Table 15-22_CN”*

**References**

1. First MB, Spitzer RL, Gibbon M, Williams JB. Structured clinical interview for DSM-IV clinical version (SCID-I/CV). Washington DC: American Psychiatric Press1997.

2. Salzman S, Endicott J, Clayton P, Winokur G. Diagnostic evaluation after death (DEAD). *National Institute of Mental Health, Rockville* 1983.

3. Donati RJ, Dwivedi Y, Roberts RC, Conley RR, Pandey GN, Rasenick MM. Postmortem brain tissue of depressed suicides reveals increased Gs alpha localization in lipid raft domains where it is less likely to activate adenylyl cyclase. *The Journal of neuroscience : the official journal of the Society for Neuroscience* 2008; **28**(12)**:** 3042-3050.

4. A Unique Approach to Lifespan Diagnosis: The Psychological Autopsy,. <https://psychiatry.pitt.edu/unique-approach-lifespan-diagnosis-psychological-autopsy>, 2020, Accessed Date Accessed 2020 Accessed.

5. Aryee MJ, Jaffe AE, Corrada-Bravo H, Ladd-Acosta C, Feinberg AP, Hansen KD *et al.* Minfi: a flexible and comprehensive Bioconductor package for the analysis of Infinium DNA methylation microarrays. *Bioinformatics* 2014; **30**(10)**:** 1363-1369.

6. Lehne B, Drong AW, Loh M, Zhang W, Scott WR, Tan ST *et al.* A coherent approach for analysis of the Illumina HumanMethylation450 BeadChip improves data quality and performance in epigenome-wide association studies. *Genome Biol* 2015; **16**(1)**:** 37.

7. Elliott HR, Tillin T, McArdle WL, Ho K, Duggirala A, Frayling TM *et al.* Differences in smoking associated DNA methylation patterns in South Asians and Europeans. *Clin Epigenetics* 2014; **6**(1)**:** 4.
